# Transcriptome Analysis of the Ovaries of Taihe Black-Bone Silky Fowls at Different Egg-Laying Stages

**DOI:** 10.3390/genes13112066

**Published:** 2022-11-08

**Authors:** Xin Xiang, Xuan Huang, Jianfeng Wang, Haiyang Zhang, Wei Zhou, Chunhui Xu, Yunyan Huang, Yuting Tan, Zhaozheng Yin

**Affiliations:** 1 Zijingang Campus, Animal Science College, Zhejiang University, Hangzhou 310058, China; 2 Hangzhou Original Seed Farm, Hangzhou 311115, China

**Keywords:** Taihe black-bone silky fowl, reproductive performance, transcriptome, ovary

## Abstract

The poor egg-laying performance and short peak egg-laying period restrict the economic benefits of enterprises relating to the Taihe black-bone silky fowl. Ovaries are the main organ for egg production in poultry. Unlike that of mammals, the spawning mechanism of poultry has rarely been reported. As a prominent local breed in China, the reproductive performance of Taihe black-bone silky fowls is in urgent need of development and exploitation. To further explore the egg-laying regulation mechanism in the different periods of Taihe black-bone silky fowls, the ovarian tissues from 12 chickens were randomly selected for transcriptome analysis, and 4 chickens in each of the three periods (i.e., the pre-laying period (102 days old, Pre), peak laying period (203 days old, Peak), and late laying period (394 days old, Late)). A total of 12 gene libraries were constructed, and a total of 9897 differential expression genes (DEGs) were identified from three comparisons; the late vs. peak stage had 509 DEGs, the pre vs. late stage had 5467 DEGs, and the pre vs. peak stage had 3921 DEGs (pre-stage: pre-egg-laying period (102 days old), peak-stage: peak egg-laying period (203 days old), and late-stage: late egg-laying period (394 days old)). In each of the two comparisons, 174, 84, and 2752 differentially co-expressed genes were obtained, respectively, and 43 differentially co-expressed genes were obtained in the three comparisons. Through the analysis of the differential genes, we identified some important genes and pathways that would affect reproductive performance and ovarian development. The differential genes were *LPAR3*, *AvBD1*, *SMOC1*, *IGFBP1*, *ADCY8*, *GDF9*, *PTK2B*, *PGR,* and *CD44*, and the important signaling pathways included proteolysis, extracellular matrices, vascular smooth muscle contraction, the NOD-like receptor signaling pathway and the phagosome. Through the analysis of the FPKM (Fragments Per Kilobase of exon model per Million mapped fragments) values of the genes, we screened three peak egg-laying period-specific expressed genes: IHH, INHA, and CYP19A1. The twelve genes and five signaling pathways mentioned above have rarely been reported in poultry ovary studies, and our study provides a scientific basis for the improvement of the reproductive performance in Taihe black-bone silky fowls.

## 1. Introduction

Egg production is an important indicator of the reproductive performance of chickens and affects the profitability and productivity of the egg industry. Eggs are inexpensive and contain a large number of nutrients (including protein, vitamins, and minerals) needed by the human body, making them important food resources for humans. Therefore, the improvement of egg production is crucial for the whole hen-egg industry [1]. The ovaries are an important female reproductive organ. Its main roles are to secrete progesterone and estrogen, produce oocytes, and promote follicle discharge. In recent years, most studies have focused on follicular development in mammals, and less on follicular development in poultry. Follicular development in poultry is a complex physiological process similar to that of mammals but with its own characteristics [2]. Hens have 5–6 stratified follicles (F1-F6) of different sizes in their ovaries, the largest follicle (F1) subsequently undergoes ovulation, and only very few follicles develop and ovulate [3]. Ovarian follicular development plays a very important role in egg production and involves various paracrine, autocrine, and endocrine factors. Egg production is a complex physiological process. In brief, eggs develop from follicles and are expelled through the ovaries. Egg formation in hens is mainly influenced by the environment and the hypothalamic-pituitary-gonadal (HPG) axis, which secretes a variety of sex hormones to maintain the normal physiological functions of the ovary [4]. There is no doubt that the normal development of ovaries and the maintenance of physiological functions are all crucial for egg production and deserve to be explored and studied.

Traditional breeding strategies require long-term selection, which is usually time-consuming and laborious. Therefore, various high-throughput sequencing technologies based on the identification of genes at the transcriptome level are increasingly used in poultry research [5]. RNA sequencing (RNA-seq) is a transcriptome analysis method that involves a sequencing analysis that employs high-throughput sequencing technology. It allows differential gene analysis at the genome-wide level and has high sensitivity [6]. Many transcriptome studies have been performed in poultry using RNA-seq. Mu et al. identified 142 differentially expressed genes in the ovaries of low-yield and high-yield Changshun green shell-laying hens, of which 55 were upregulated, and 87 were downregulated. They predicted that the *PRLR*, *NRP1*, *IL15*, *BANK1*, *NTRK1*, *CCK,* and *HGF* genes would be involved in regulating egg production and identified two associated pathways; the neuroactive ligand-receptor interaction and transmembrane receptor protein-tyrosine kinase activity [1]. Tao et al. identified 843 differentially expressed genes in the ovaries of high-yield and low-yield Jinding ducks, of which 476 were upregulated and 367 were downregulated. They predicted that the *MC5R*, *APOD*, *ORAI1,* and *DYRK4* genes would play important roles in regulating egg production and identified several important pathways, including the steroid biosynthesis pathway, fatty acid biosynthesis, and calcium-signaling pathway [7].

The Taihe black-bone silky fowl is a local Chinese breed that originated in Wangbantu village, Taihe county, Jiangxi province. It has a high special economic value and is considered by traditional medicine as a precious species of poultry with both medicinal and food uses unique to China [8]. In addition, it has high ornamental value. However, the poor egg-laying performance and short peak egg-laying period restrict the economic benefits of enterprises relating to this type of chicken. RNA-seq is widely used for transcriptome sequencing, which can discover genes that have a large impact on poultry egg production performance, reveal key pathways and candidate genes that control egg production rates, and provides a theoretical basis for regulating poultry egg production performance at the molecular level. We provide new insights into the epigenetic regulation mechanisms of egg production performance in the Taihe black-bone silky fowl.

To explore the differentially expressed genes and pathways that affect egg production performance, we selected three periods: the pre-egg-laying period, the peak egg-laying period, and the late egg-laying period. Using transcriptome sequencing, we explored the differential genes and pathways in different laying periods, and by comparing the three periods, we screened out the genes and pathways that have significant effects on egg production performance. In this study, RNA-seq technology was used to identify the differentially expressed genes in the ovaries of Taihe black-bone silky fowls at different egg-laying stages, and the molecular mechanisms involved in egg-laying performance were revealed through the analysis of the differential genes and related pathways. These results provide a new perspective on regulating ovarian development in Taihe black-bone silky fowls while offering a basis for improving their egg production performance.

## 2. Materials and Methods

### 2.1. Animal and Sample Collection

Twelve Taihe black-bone silky fowls (four 102-day-old chickens, four 203-day-old chickens, and four 394-day-old chickens) were purchased from the Taihe county in the Jiangxi province from the Taihe Aoxin black-bone silky fowl Development Co (Taihe county, Jiangxi province, China). The sample chickens were randomly selected and kept under the same feeding conditions. The ovarian tissues from these 12 chickens were collected and immediately preserved in liquid nitrogen.

### 2.2. Ethical Statement

All animal experiments conformed to the standards in the Chinese animal welfare guidelines and were approved by the Animal Experimentation Ethics Committee of Zhejiang University (approval number: ZJU20190149).

### 2.3. Library Construction and Sequencing of the Transcriptome

The ovarian tissues collected from the hens were sent to Beijing Novozymes Technology Co., Ltd., who was responsible for the construction of the library and sequencing. Subsequently, the libraries were constructed and sequenced on an Illumina NovaSeq 6000.

### 2.4. Bioinformatics Analysis of the Transcriptome

To ensure the quality and reliability of the data analysis, the raw data needed to be filtered to obtain clean data (clean reads). Meanwhile, the content of Q20, Q30, and GC was calculated using the clean data. All subsequent analyses were based on high-quality clean data. The reference genome and gene model annotations were downloaded directly from the genome website (http://ftp.ensembl.org/pub/release-105/fasta/gallus_gallus/, http://ftp.ensembl.org/pub/release-105/gtf/gallus_gallus/, accessed on 15 August 2022). The index of the reference genome was constructed using Hisat2 v2.0.5 while comparing the clean reads from the paired ends to the reference genome. Featurecounts (1.5.0-p3) were used to calculate the reads and FPKM mapped to each gene.

### 2.5. Differential Expression Analysis

Differential expression analysis was performed on four biological replicates using DESeq2 (1.20.0). Based on the model of the negative binomial distribution, DESeq2 identified the differential expression in the gene expression data by using a statistical procedure. The resulting *p*-values were adjusted using the Benjamini-Hochberg method to control for the false discovery rate [9]. The genes with *p* values of less than 0.05 found by DESeq2 were assigned as differentially expressed genes.

### 2.6. Analysis of the GO and KEGG Pathways

The GO enrichment analysis of the differentially expressed genes was performed by clusterProfiler (3.8.1) software, and the gene length bias was adjusted. The differentially expressed genes were significantly enriched for GO keywords with *p*-values less than 0.05.

KEGG is a data resource library which is used to understand the advanced functions and effectiveness of biological systems (such as cells, organisms, and ecosystems) from molecular-level information. It is primarily used for large-scale molecular datasets and high-throughput databases. To analyze the statistical enrichment of differentially expressed genes in the KEGG pathway, we used clusterProfiler (3.8.1) software.

## 3. Results

### 3.1. Sequencing Results and Reading Mapping

A total of 511,570,530 clean reads were obtained from 12 libraries, with an average of 42,630,878 clean reads per sample (the number of reads ranged from 40,271,656 to 48,173,986). The Q20 content ranged from 96.89% to 97.3%, the Q30 content ranged from 92.21% to 92.85%, and the GC content ranged from 48.16% to 52.02%. Meanwhile, the reads mapped to the chicken genome all exceeded 90% (Table 1).

### 3.2. Differential Expression Genes

**Figure 1:**
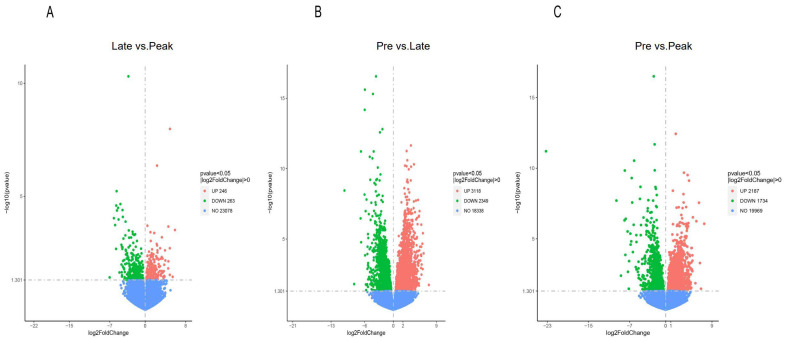
The volcano plot of the DEGs (differential expression genes) in the (**A**) late vs. peak stage, (**B**) pre vs. late stage, and (**C**) pre vs. peak stage. The X-axis means the change of gene multiplicity (log2FoldChange). The Y-axis indicates the significance level of the difference (−log10pvalue). Red dots: upregulated genes; green dots: downregulated genes; blue dots: non-differential genes.

**Figure 2:**
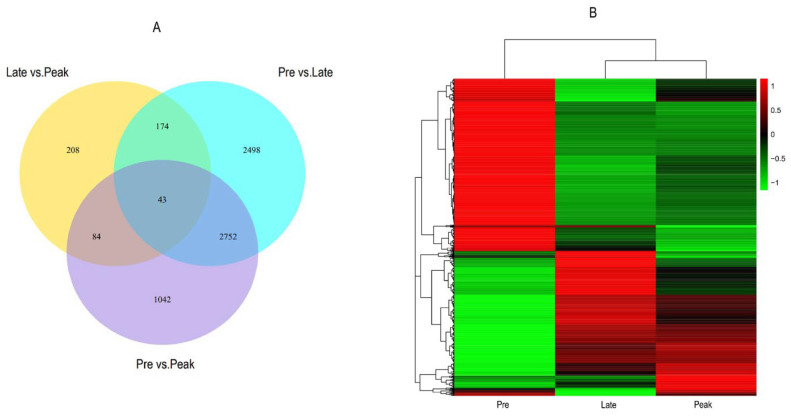
Venn diagram of the three comparisons of the differentially expressed genes (**A**). Hierarchical clustering analysis of the DEGs in the three comparisons (**B**).

A total of 9897 differential expression genes (late vs. peak: 509 DEGs, pre vs. late: 5467 DEGs, and pre vs. peak: 3921 DEGs) were obtained for the three comparisons; 246 upregulated genes and 263 downregulated genes were discovered in the late vs. peak stage, 3118 upregulated genes and 2349 downregulated genes were discovered in the pre vs. late stage, and 2187 upregulated genes and 1734 downregulated genes were discovered in the pre vs. peak stage. In addition, 174, 84, and 2752 differentially co-expressed genes were obtained from two comparisons, and 43 differentially co-expressed genes were obtained from three comparisons. We screened nine differential genes in the reproduction-related pathways that may affect the egg-laying performance of Taihe black-bone silky fowls, i.e., *LPAR3*, *AvBD1*, *SMOC1*, *IGFBP1*, *ADCY8*, *GDF9*, *PTK2B*, *PGR,* and *CD44*. Furthermore, we screened three peak egg-laying period-specific expressed genes from the FPKM values, i.e., *IHH*, *INHA,* and *CYP19A1*. (Figure 1 and Figure 2, Tables S1 and S2, and Figure S1).

### 3.3. Gene Ontology Enrichment Analysis

**Figure 3:**
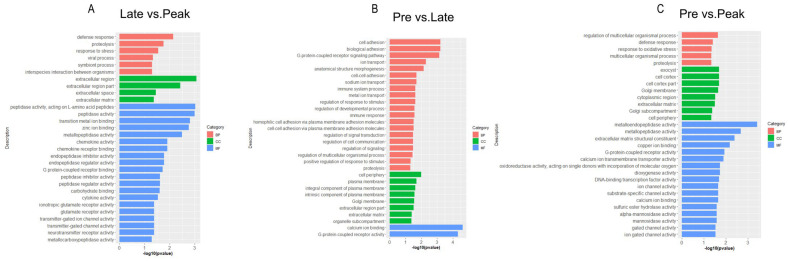
The GO enrichment bar graphs for the top 30 items in the (**A**) late vs. peak stage, (**B**) pre vs. late stage, and (**C**) pre vs. peak stage. (BP: biological process, CC: cellular component, MF: molecular function).

In order to better understand the process of ovarian development, we performed a GO analysis on the DEGs from three comparison groups. GO is a comprehensive database describing gene functions, which can be divided into three parts: biological processes (BPs), cellular components (CCs), and molecular functions (MFs). In the late vs. peak stage, a total of 336 DEGs were enriched to 402 terms, including 186 BPs, 42 CCs, and 174 MFs. The most significantly enriched terms included defense response, proteolysis, extracellular matrices, zinc ion binding, chemokine activity, and chemokine receptor binding. In the pre vs. late stage, a total of 3423 DEGs were enriched to 877 terms, containing 459 BPs, 125 CCs, and 293 MFs. The most significantly enriched terms included cell adhesion, the G-protein-coupled receptor signaling pathway, calcium ion binding, proteolysis and extracellular matrices. In the pre vs. peak stage, a total of 2544 DEGs were enriched into 824 terms, containing 408 BPs, 120 CCs, and 296 MFs. The most significantly enriched terms included the regulation of the multicellular organismal process, proteolysis, extracellular matrices, metallopeptidase activity, G-protein-coupled receptor activity, and calcium ion transmembrane transporter activity. In all three comparison groups, proteolysis and extracellular matrices were significantly enriched (Figure 3 and Table S3).

### 3.4. KEGG Pathway Analysis

**Figure 4:**
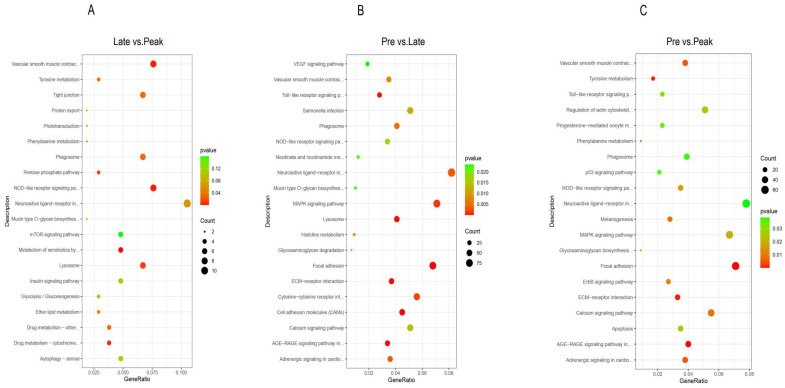
The 20 most significant KEGG pathways in the three comparison groups. (**A**): the late vs. peak stage, (**B**): the pre vs. late stage, and (**C**): the pre vs. peak stage.

To gain more insight into the process of ovarian development, we performed a KEGG pathway analysis on the DEGs from the three comparison groups. KEGG (Kyoto encyclopedia of genes and genomes) is a comprehensive database that integrates genomic, chemical, and systematic functional information. In the late vs. peak stage, 105 DEGs were enriched in 105 pathways, of which 11 were significantly enriched, mainly in the metabolism of xenobiotics by cytochrome P450, vascular smooth muscle contraction, the NOD-like receptor signaling pathway, and the phagosome and lysosome. In the pre vs. late stage, 1096 DEGs were enriched in 151 pathways, of which 29 were significantly enriched, mainly in the ECM-receptor interaction, focal adhesion, vascular smooth muscle contraction, NOD-like receptor signaling pathway, and phagosome. In the pre vs. peak stage, 818 DEGs were enriched in 151 pathways, of which 22 were significantly enriched, mainly in focal adhesion, the AGE-RAGE signaling pathway in diabetic complications, vascular smooth muscle contraction, and the NOD-like receptor signaling pathway and phagosome. The pathways that were significantly enriched in all three comparisons were vascular smooth muscle contraction, the NOD-like receptor signaling pathway, and the phagosome (Figure 4 and Table S4).

## 4. Discussion

Improving egg production is an important goal for the poultry industry. Ovarian health and normal ovulation are very important for poultry production. By studying the expression patterns of ovarian genes during development, we could help improve the reproductive performance and better understand the physiological performance of the Taihe black-bone silky fowl. Hu et al.’s ovarian transcriptomic analysis of the black Muscovy duck at the early, peak, and late egg-laying stages found five genes involved in ovarian development, *HOXA10*, *HtrA3*, *StAR*, *ZP2* and *TAT*, as well as two important pathways that may be associated with egg production, namely steroid hormone biosynthesis and ovarian steroidogenesis [10]. Zhang et al. predicted four genes affecting egg production, *FOXA2*, *MED37D*, *HSP70* and *RXFP2*, and three pathways that may be associated with egg production, namely the longevity regulating pathway-multiple species pathway, the estrogen signaling pathway and the PPAR signalling pathway, in the comparative transcriptomic analysis of the ovaries from high and low egg-laying Lingyun black-bone chickens [11]. In this study, we predicted twelve genes that may affect reproductive performance, *LPAR3* (lysophosphatidic acid receptor 3), *AvBD1* (avian β-defensin 1), *SMOC1* (SPARC-related modular calcium binding 1), *IGFBP1* (insulin-like growth factor binding protein 1), *ADCY8* (adenylyl cyclase type 8), *GDF9* (growth differentiation factor 9), *PTK2B* (protein-tyrosine kinase 2 beta), *PGR* (progesterone receptor), *CD44* (cluster of differentiation-44), *IHH* (Indian hedgehog), *INHA* (inhibin α), and *CYP19A1* (cytochrome P450 family 19 subfamily A member 1), and five pathways: proteolysis, extracellular matrices, vascular smooth muscle contraction, and the NOD-like receptor signaling pathway and phagosome.

### 4.1. Analysis of DEGs

Lysophosphatidic acid (LPA) is a growth factor-like phospholipid that regulates cell growth, proliferation, differentiation, and intracellular messaging by activating multiple G protein-coupled receptors [12]. *LPAR3* belongs to the LPA family, which is used to encode LPA3, the third G protein-coupled receptor for lysophosphatidic acid (LPA) [13]. *LPAR3* can interact with reproductive hormones to affect reproductive performance, including estrogen and progesterone [14]. Several studies have shown that *LPAR3* enhances nitric oxide levels in the non-pregnant buffalo uterus and facilitates the maintenance of the corpus luteum [15]. This gene affects reproduction in mice by regulating the spacing of embryos [16]. In addition, it can affect human embryo implantation and endometrial decidualization [17]. Avian β-defensin 1 (*AvBD1*) is a member of the avian β-defensin family, which is an important component of the immune system. It was shown that *AvBD1* has a protective effect on the development of chick embryos [18]. *AvBd1* is expressed in the reproductive organs of hens, which can effectively resist pathogenic microorganisms, maintain the normal development of the ovaries, and affect the laying performance of hens [19]. In addition, *AvBD1* is expressed in the theca and granulosa layers, which can affect the innate immune system of the ovaries and has a regulatory role in normal ovarian development [20]. SPARC-related modular calcium binding 1 (*SMOC1*) is an extracellular calcium-binding protein of the BM-40 family that is mainly expressed in organ basement membranes [21]. *SMOC1* may mediate intercellular signaling and cell type-specific differentiation during fetal gonad and fetal reproductive tract development [22]. It was reported that *SMOC1* may have an important regulatory role in the egg production performance of Moscow ducks [23]. Moreover, *SMOC1* is highly expressed in ovarian and embryonic development and has a role in ovarian development and maintenance [24]. In summary, *LPAR3*, *AvBD1,* and *SMOC1* were significantly upregulated in the peak group compared with the late group, suggesting that *LPAR3*, *AvBD1,* and *SMOC1* may have important roles in egg production performance during the peak period of egg production.

Insulin-like growth factor binding protein 1 (*IGFBP1*) is a multifunctional protein involved in various physiological processes, such as growth, development, and reproduction. It was found to be a marker of the endometrium and can be used for the screening of female polycystic ovary syndrome [25]. *IGFBP1* contributes to the ectodermal migration and attachment to the endometrium [26]. Several studies have shown that the increased levels of *IGFBP1* expression facilitate the differentiation of endometrial stromal cells and have an important role in the establishment of pregnancy [27,28]. In addition, it allows the genetic diagnosis of hypertension in pregnancy [29]. *ADCY8* is a member of the adenylate cyclase family and constitutes only a minor adenylated cyclic glucosyl methyl ether [30,31]. *ADCY8* can maintain the HPG axis of zebrafish and is very important for zebrafish reproduction [32]. It was shown that *ADCY8* plays a key role in the spawning rate of ducks [33]. In addition, *ADCY8* is mainly involved in the production of ovarian steroids, which have a regulatory effect on ovarian development and estrogen [34]. Growth differentiation factor 9 (*GDF9*) is an oocyte secretory factor which regulates egg quality and developmental capacity and has a dominant role in ovarian function [35]. It was shown that abnormalities in *GDF9* could lead to the development of human ovarian diseases [36]. *GDF9* can promote follicular development and maintain the zona pellucida structure in mice [37,38]. Decreased levels of *GDF9* expression could affect ovarian and follicular development and lead to premature ovarian failure [39]. In addition, *GDF9* has the potential to improve female infertility [40]. In the present study, *IGFBP1*, *ADCY8,* and *GDF9* were significantly upregulated in the pre group compared to the late group. The results suggest that these three genes may contribute to egg production performance in the pre-laying period and may play a regulatory role in follicle development and ovarian maintenance.

Protein-tyrosine kinase 2 beta (*PTK2B*) is a member of the focal adhesion kinase family and plays a key role in oocyte maturation and in vitro fertilization [41,42]. Sharma et al. found that *PTK2B* is expressed in zebrafish oocytes and affects zebrafish reproduction [43]. Meng et al. found that *PTK2B* can mediate the process of early embryonic development in mice [42]. It was reported that *PTK2B* regulates female fertility by regulating the process of ovarian and follicle formation, and when it is inhibited, oocyte maturation and ovarian development are affected [41]. In addition, *PTK2B* was found to be involved in the process of human sperm capacitation [44]. The progesterone receptor (*PGR*) plays an important role in regulating a variety of reproductive processes, including endometrial differentiation, embryonic development, and mammary gland development [45]. It can influence embryo implantation and pregnancy maintenance through estrogen [46]. Kim et al. found that mice were infertile due to a loss of *PGR* signaling [47]. The progesterone receptor (*PGR*) is highly expressed in the ovaries and is essential for successful ovulation. In addition, it can regulate the physiological function and morphology of the fallopian tubes and affect oocyte maturation and fertilization [48]. It has been shown that *PGR* can mediate the inflammatory response of the ovaries during ovulation and has a regulatory role in the normal development of the ovaries [49]. *CD44* is a cell surface adhesion molecule that binds mainly to hyaluronic acid and is a ubiquitous glycoprotein [50,51]. Paravati et al. found that *CD44* and osteopontin (OPN) form protein complexes, which are important materials for embryonic recognition and can also mediate endometrial receptivity [52]. Moreover, *CD44* can also promote blastocyst attachment. *CD44* is an important mediator of progesterone and estradiol and an important gene marker of cumulus cells in oocytes [53,54,55]. We concluded that *CD44* has a regulatory role in ovarian development and the normal egg production of laying hens. In this study, *PTK2B*, *PGR,* and *CD44* were significantly upregulated in the peak group compared with the pre group, suggesting that these three genes are involved in the regulation of peak egg production and may play an important role in ovarian maintenance and the promotion of oocyte maturation.

Indian hedgehog (*IHH*) is a member of the hedgehog (HH) family and plays an important regulatory role in ovarian development. It activates the HH pathway in the ovary, regulates steroid hormones in the ovary, and maintains normal ovarian development [56]. It was shown that *IHH* deficiency could lead to ovarian dysplasia, which could be harmful to women’s reproductive health [57]. In addition, *IHH* is an important mediator of progesterone signal transduction in the uterus and plays an important role in embryo implantation [58]. Inhibin α (*INHA*), a member of the transforming growth factor-β (TGF-β) superfamily, plays an important role in the regulation of reproduction, such as folliculogenesis, oocyte maturation, and embryonic development [59,60]. Inhibin can regulate the release of FSH from pituitary cells, and the inactivation of inhibin leads to elevated FSH levels, ovarian hyperstimulation, and abortion in female mice [61]. *INHA* was reported to be a potential regulator of ovarian egg production in Luhua chickens [62]. We speculated that this gene has an important role in the egg production performance of hens. Cytochrome P450 family 19 subfamily A member 1 (*CYP19A1*) is expressed in granulosa cells of mammalian ovaries. Unlike mammals, *CYP19A1* is mainly expressed in the theca cells of hen ovary follicles [63]. In birds, *CP19A1* can mediate estrogen production as a way to affect normal ovarian development and egg production [64]. The expression of the three genes was significantly higher in the peak-laying period than in the pre-laying and late-laying periods, and we speculate that the three genes have an important role in normal ovarian development and egg production performance.

### 4.2. Analysis of GO and KEGG

In order to further understand the functions that may be involved in the ovarian development of Taihe black-bone silky fowls, we performed GO annotations (gene ontology) and KEGG analyses (Kyoto encyclopedia of genes and genomes) on the DEGs. The GO enrichment results of the three comparisons showed that proteolysis and the extracellular matrix were significantly enriched in all three comparison groups. Proteolysis is an irreversible post-translational modification process characterized by the highly precise and stable cleavage of proteins. Downstream events in the signaling process depend on the triggering of proteolysis by protease activity [65]. Some studies have shown that in mammalian ovaries, the proteolysis of the extracellular matrix is dynamically regulated by plasminogen activator and plasminogen activator inhibitor (PAI) and is a key event affecting various physiological and pathological processes. Proteolysis is an important part of the ovulation process. Proteolysis in the follicle wall can mediate the rupture of pre-ovulation follicles and release mature oocytes [66,67,68]. Bentov et al. found that proteolysis affects the estrogen concentration in follicles [69]. In addition, there is a link between proteolysis and estrogen receptor activation [70]. The extracellular matrix is present in all organs and tissues and is a mixture of cellular and non-cellular components that can regulate a variety of cellular processes [71]. It was shown that the extracellular matrix is a major component of the ovarian matrix and can mediate many physiological functions in the ovary, such as follicular growth and development, ovulation, and steroidogenesis [72]. The enrichment of the extracellular matrix is consistent with a recent report on Nandan-Yao domestic chickens [73]. Zhou et al. found that the extracellular matrix could directly affect the follicle growth of chickens [74]. Fukuda et al. found that the extracellular matrix may affect the formation of primordial follicles in mice [75]. In addition, it was shown that the cumulus matrix is essential for in vivo fertilization [76]. Calcium ion binding and G-protein-coupled receptor activity are the greatest contributors to the molecular functional enrichment of DEGs, and they may play a crucial role in the egg production of Taihe black-bone silky fowls.

For the KEGG signaling pathway, the pathways that were significantly enriched in all three comparison groups were vascular smooth muscle contraction, the Nod-like receptor signaling pathway, and the phagosome. Vascular smooth muscle (VSM) plays a key role in the regulation of vascular function, and calcium ions are the main regulator of VSM contraction [77]. It was shown that progesterone can affect VSM contraction by reducing intracellular calcium levels through signaling pathways (mainly MAP and Akt pathways); progesterone was shown to affect endometrial differentiation and promote oocyte maturation [78,79]. Lu et al. found that in pigeon follicles, DEGs were significantly enriched in the vascular smooth muscle contraction pathway, promoting follicle maturation and ovulation [80]. Therefore, we speculate that the vascular smooth muscle contraction pathway could indirectly affect ovarian maintenance and promote oocyte maturation and follicle development. The NOD-like receptor signaling pathway plays an important role in the immune response, which can convert danger signals into pro-inflammatory cytokines and chemokines; some studies have shown that chemokines are expressed in oocytes and follicles and may affect follicular and reproductive functions. Chemokines bind to their receptors and also affect endometrial physiology [81,82]. Therefore, we speculate that the Nod-like receptor signaling pathway may indirectly affect ovarian function. Jiang et al. found that the Nod-like receptor signaling pathway affects the pyrophosphorylation of ovarian granulosa cells, which further affects ovarian function [83]. In addition, Martyniak et al. found that the NOD-like receptor signaling pathway may have a regulatory role in the oviduct and may affect the maturation of oocytes [84]. The phagosome is essential in adaptive and innate immunity. Several studies have shown that the phagosome pathway affects the physiological function of luteal cells and influences subsequent ovulation. Furthermore, it is positively correlated with follicular development [85]. Candelaria et al. found that the phagosome pathway can affect follicle-stimulating hormone production [86]. The absence of caseinolytic peptidase P leads to female infertility and accelerated follicular depletion, while the absence of caseinolytic peptidase P significantly affects the phagosome pathway [87]. Therefore, we speculate that the phagosome pathway may affect female reproductive function. In summary, in our study, vascular smooth muscle contraction, the Nod-like receptor signaling pathway, and the phagosome were significantly enriched in all three comparison groups. Although they have rarely been reported, they may play very important roles in the reproduction and egg production of Taihe black-bone silky fowls. In general, our study provides some new insights into the pathways associated with egg production in poultry.

## 5. Conclusions

In this study, we detected a total of 9897 differential expression genes (late vs. peak: 509 DEGs, pre vs. late: 5467 DEGs, and pre vs. peak: 3921 DEGs) potentially associated with egg production performance via the transcriptome sequencing analysis of the ovaries of Taihe black-bone silky fowls at different egg-laying periods. Furthermore, we identified twelve key genes that may affect egg production performance: *LPAR3*, *AvBD1*, *SMOC1*, *IGFBP1*, *ADCY8*, *GDF9*, *PTK2B*, *PGR*, *CD44*, *IHH*, *INHA,* and *CYP19A1*. Based on the GO and KEGG enrichment analysis, we initially explored five pathways that could affect egg production performance: proteolysis, extracellular matrices, vascular smooth muscle contraction, the NOD-like receptor signaling pathway, and the phagosome. These genes and pathways may play a decisive role in improving the egg production performance of Taihe black-bone silky fowls. We compared the gene expression levels of the ovarian tissues of Taihe black-bone silky fowls in different egg-laying periods with RNA-seq, and laid the foundation for subsequent research on the screening of candidate functional genes and gene function verification of important production traits in Taihe black-bone silky fowls. Lastly, we provide a theoretical basis for the development of molecular breeding to improve the egg production performance of Taihe black-bone silky fowls.

## Figures and Tables

**Table 1:** Sequencing data.

Sample	Raw Reads	Clean Reads	Total Map	Q20	Q30	GC pct
Pre_1	45,325,812	41,798,666	38,517,440 (92.15%)	97.3%	92.85%	50.26%
Pre_2	52,044,340	48,173,986	43,445,903 (90.19%)	96.89%	92.24%	48.89%
Pre_3	44,553,380	40,271,656	36,994,662 (91.86%)	97.18%	92.71%	50.83%
Pre_4	45,781,184	41,751,192	37,993,982 (91.0%)	96.95%	92.27%	48.16%
Peak_1	47,304,680	43,650,052	39,559,715 (90.63%)	96.95%	92.21%	50.97%
Peak_2	43,973,992	40,910,080	37,503,629 (91.67%)	97.21%	92.76%	50.4%
Peak_3	46,869,756	43,034,208	39,548,737 (91.9%)	97.21%	92.76%	50.44%
Peak_4	49,189,518	45,234,226	41,466,481 (91.67%)	97.14%	92.61%	50.54%
Late_1	45,141,060	41,969,306	38,109,595 (90.8%)	96.99%	92.34%	52.02%
Late_2	44,914,334	41,107,798	37,444,801 (91.09%)	97.18%	92.71%	51.4%
Late_3	43,823,110	40,425,332	36,598,028 (90.53%)	97.1%	92.62%	51.44%
Late_4	47,357,330	43,244,028	39,397,767 (91.11%)	97.09%	92.5%	50.8%

## Data Availability

The sequence data were submitted to the NCBI SRA database under the accession number PRJNA889190.

## Supplementary Materials

The following supporting information can be downloaded at: https://www.mdpi.com/article/10.3390/genes13112066/s1, Figure S1: MA-plot; Table S1: List of all DEGs of the three comparison groups; Table S2: The expression level of important genes(fpkm); Table S3: List of GO terms of the three comparison groups; Table S4: List of KEGG pathways of the three comparison groups.
